# Reduced Cerebral Infarct Volume in Young UCP2^−/−^ Mice and Preserved Synaptic Transmission by Genipin

**DOI:** 10.3390/cells15141299

**Published:** 2026-07-21

**Authors:** Gesine Reichart, Henrieke Koch, Tina Sellmann, Anne Einsle, Johannes Mayer, Robert Jaster, Timo Kirschstein, Falko Lange, Rüdiger Köhling

**Affiliations:** 1 Oscar-Langendorff-Institute of Physiology, Rostock University Medical Center, 18057 Rostock, Germany; henrieke.koch@uni-rostock.de (H.K.); tina.sellmann@uni-rostock.de (T.S.); anne.einsle@med.uni-rostock.de (A.E.); johannes.mayer@medicalschool-hamburg.de (J.M.); timo.kirschstein@uni-rostock.de (T.K.); falko.lange@uni-rostock.de (F.L.); ruediger.koehling@uni-rostock.de (R.K.); 2 Department of Human Medicine, MSH Medical School Hamburg, 20457 Hamburg, Germany; 3 Department of Internal Medicine, Division of Gastroenterology, Hepatology and Nutritional Medicine, Rostock University Medical Center, 18057 Rostock, Germany; robert.jaster@med.uni-rostock.de

**Keywords:** ischemic stroke, oxidative stress, uncoupling protein 2, middle cerebral artery occlusion, oxygen-glucose deprivation, genipin

## Abstract

**Highlights:**

**What are the main findings?**

- After transient occlusion of the middle cerebral artery, UCP2^−/−^ mice at the age of 6 months had a reduced infarct volume, mainly in the core area.
- UCP2 inhibitor genipin preserved synaptic transmission in the hippocampal formation under oxygen-glucose deprivation.

**What are the implications of the main findings?**

- Mild persistent oxidative stress may help to diminish ischemia-reperfusion injury.
- Genipin may act in a neuroprotective manner under hypoxia and reperfusion.

**Abstract:**

Cerebral ischemia–reperfusion injury is a key determinant of a poor outcome after stroke. The mitochondrial uncoupling protein 2 (UCP2) has been implicated in cerebral ischemia-reperfusion injury and in the outcome of ischemic stroke, although its role remains controversial. In C57BL/6J and B6.129S4-*Ucp2*^tm1Lowl/J^ (UCP2^−/−^) mice, we analyzed cognitive function and lifespan. In an MCAO model induced for one hour, infarct volumes, neurological deficits, and gene expression patterns were determined after 24 h. The UCP2 inhibitor genipin was used in an oxygen-glucose deprivation (OGD) model to investigate synaptic transmission in the hippocampus. Compared to controls, UCP2^−/−^ mice exhibited a reduced lifespan and displayed impaired cognition. However, in 6-month-old UCP2^−/−^ mice, the infarct volume was reduced, primarily due to a smaller core size, but not in 18-month-old animals. In both strains, ischemia induced upregulation of antioxidant defense genes, including catalase and SOD1. In the ex vivo ODG model, synaptic transmission was depressed, but pretreatment with genipin prevented the tissue from this impairment. Our findings indicate an infarct-reducing effect of UCP2 deficiency, especially in young-adult mice, and, mechanistically, a neuroprotective effect by genipin in hippocampal slices.

## 1. Introduction

Cerebral ischemic stroke ranks among the highest causes of death in the world, and it puts a major burden on neurological healthcare in elderly populations [1]. If the diagnosis is made in time, recanalization of the hypoperfused areas through thrombolysis is pursued. However, only 10–15% of patients meet the necessary requirements to offer recanalization procedures [2]. Recent multicenter randomized clinical trials and meta-analyses indicate that in up to 60% of patients, recanalization is futile, with no or minimal improvement in functional outcome [3]. A major contributor to this limited efficacy is ischemia-reperfusion injury, a complex and multifactorial pathophysiological process triggered upon restoration of the blood flow [4,5].

One key player that highly contributes to cerebral ischemia-reperfusion injury is oxidative stress, primarily based on reactive oxygen species (ROS) [4,6]. Excessive levels of ROS may accrue not only in the state of high metabolic activity during the process of reperfusion after ischemia but also to a lesser extent in the hypoperfused phase. Due to the high energy demand of the cells after recanalization, overwhelming levels of ROS accumulate in the mitochondria that may lead to tissue damage based on lipid peroxidation, protein denaturation, inflammation, excitotoxicity, and apoptosis [6,7]. During ischemia, oxidative stress may arise from ROS release by mitochondria, often due to an overburden of the antioxidant defense impaired by the energy depletion in the cells [8,9,10].

One regulator of ROS is the uncoupling protein 2 (UCP2), a member of the UCP family localized in the inner mitochondrial membrane. So far, its physiological functions are only incompletely understood. Primarily, it functions as a proton carrier and contributes to the mitochondrial proton leak [11], but additional functions such as transporting C4 metabolites from the matrix of mitochondria (and thereby preventing oxidative stress) were found in other investigations [12,13,14].

Previous studies had shown that UCP2 may counter mild levels of oxidative stress [15]. In ischemic lesions, UCP2 expression was found to be rapidly upregulated, underlining its value in the regulation of ROS [16]. So far, in pre-clinical models, the role of UCP2 in ischemic stroke was investigated mostly in UCP2 knockout mice. In wild-type murine brains, UCP2 is expressed in a heterogeneous pattern [17,18], and expression may vary with age [19]. A lack of functional UCP2 often exacerbates neuronal deficits and reduced survival of the animals after ischemic conditions. The effects of the UCP2 knockout were linked to induction of apoptosis, ferroptosis, and neuroinflammation [20,21,22].

In marked contrast, investigations from de Bilbao and colleagues showed resistance to cerebral ischemic injury and neuroprotective effects such as increased ROS defense in UCP2 knockout mice [23]. Zhao et al. treated mice with genipin, a UCP2 inhibitor [24,25], for several days prior to ischemic infarction [26]. In the latter, inhibition of UCP2 contributed to reduced caspase activation and, eventually, a decreased infarct volume in the animals. The effects of genipin were linked to sirtuin 3 activity. While the study focused on cellular survival, functional experiments on neuronal activity were not included. In line with the concept of mitohormesis [27,28], it could be speculated that a predisposition to mild hypoxic conditions may help to protect from severe effects by subsequent infarction later on [29,30,31].

Those interesting findings encouraged us to investigate the role of UCP2 in ischemic stroke. Since previous studies limited investigations to young mice, we expanded our study to aged mice, mimicking the clinically highly relevant group of elderly patients who are more frequently affected by infarction due to mechanisms of aging [32]. To gauge the typical senile phenotype of UCP2^−/−^ mice, a lifespan analysis was initiated prior to experiments on ischemic stroke.

## 2. Materials and Methods

### 2.1. Animal Models

For in vivo experiments, 3- to 6-month-old and 18-month-old C57BL/6J (BL6) mice and B6.129S4-*Ucp2*^tm1Lowl/J^ (UCP2^−/−^) mice were used (both strains were obtained from Jackson laboratory (Bar Harbor, ME, USA)). For the in vitro experiments, 3- to 6-month-old BL6 mice served as organ donors. All experiments were conducted with both sexes and were performed according to national and international guidelines on the ethical use of animals (European Council Directive 86/609/EEC), approval of local authority Landesamt für Landwirtschaft, Lebensmittelsicherheit und Fischerei Mecklenburg-Vorpommern in Rostock, Germany (M-V/TSD/7221.3-1.1-059/12 and M-V/TSD/7221.3-1-028/17) and Animal Care and Use Committee Lübeck (V242-7224. 122-5, Kiel, Germany).

Cages were equipped with nesting material and red polycarbonate shelters to provide environmental enrichment. Animals were maintained under controlled conditions (room temperature 23 ± 2 °C, relative humidity 40 ± 5%, day-night rhythm with illumination from 6 a.m. to 6 p.m.) in groups of up to four animals per cage. Water and food were available ad libitum. For experiments, animals were randomly selected from the housing unit chosen via random numbers for experimental groups. As in our study, classification into the control or experimental group was determined by genetic background; no further randomization was feasible.

To assess lifespan in both mouse strains, a total of 60 BL6 mice (41 male, 19 female) and 60 UCP2^−/−^ mice (26 male, 34 female) were included in a longitudinal study. Animals were monitored at regular intervals by trained personnel until natural death or until predefined humane endpoints were reached. A mouse presenting more than one of the following clinical signs was determined moribund [33]: inability to eat or drink, severe lethargy (reluctance to move when gently prodded with forceps), severe balance or gait disturbance, rapid weight loss (more than 20% decrease compared to baseline for more than 3 consecutive days), an ulcerated or bleeding tumor, enlarged abdomen. Moribund mice were euthanized, and their age at death was considered the most accurate approximation of natural lifespan.

Across all experiments conducted in this study, 252 mice (BL6: *n* = 138 and UCP2^−/−^: *n* = 114) were used (Supplementary Table S1).

### 2.2. Morris Water Maze

Cognitive functions in both mouse strains at the age of 3–6 months were evaluated using an open-field version of the Morris water maze, as previously described in detail [34]. All experiments were conducted under standardized conditions, including a room temperature of 22 ± 1 °C, water temperature of 21 ± 1 °C, and illumination of approximately 110 lux, within a dedicated behavioral testing facility designed to minimize external noise. Prior to testing, animals were acclimated to the environment by being housed in the behavioral laboratory for two days. Swimming performance was recorded via video tracking and later analyzed using EthoVision 3.1 software (Noldus, Wageningen, The Netherlands). To obscure the platform, milk was added to the water, rendering it opaque. For each trial, both the latency to locate the hidden platform and the distance traveled were measured and averaged per animal per day. After seven days of training to reach the hidden platform, two additional probe trials were carried out on day eight. In these trials, the platform was removed, and the animals’ exploration was assessed over two 30-s sessions. Time spent in each of the four quadrants (target, opposite, left, and right) as well as the number of entries into the target quadrant were estimated.

Mice that completed the Morris Water Maze task at the age of 3 to 6 months were returned to their previous housing conditions and, in accordance with the 3R principles, were also utilized for lifespan analyses.

### 2.3. Focal Stroke Model—Transient Occlusion of the Middle Cerebral Artery

Transient occlusion of the middle cerebral artery (tMCAO) was applied by using the intraluminal suture-filament model derived from the Koizumi method [35]. The MCAO surgery was performed as described by Dirnagl et al. 2012 [36]. Therefore, mice were anesthetized with 2% isoflurane and maintained in 1% isoflurane with a mixture of 75% N_2_O and 25% O_2_. For tMCAO induction, the mouse was placed in a supine position on a heating plate to maintain body temperature at 37.0 ± 0.5 °C. After disinfecting the skin of the ventral neck, a midline neck incision was made. All the soft tissues were pulled apart, and the left common carotid artery (CCA) was carefully freed from connective tissue. A ligature was made 12–15 mm proximal from the carotid bifurcation using a 4.0-diameter suture thread. Then, the external carotid artery (ECA) on the same side was separated, and a second knot was made. Next, the internal carotid artery (ICA) was isolated, and a loose knot (7.0-diameter thread) was prepared around it. Proximal to this knot, the ICA was clipped to stop blood flow temporarily before a small hole was cut in the CCA about 10 mm below the bifurcation. A silicon-coated monofilament (Medium MCAO suture 70L56, Doccol^®^, Sharon, MA, USA) was introduced into the CCA to the bifurcation. Next, the clip was opened while the filament was inserted into the ICA to occlude the MCA, and the loose knot around the ICA was closed to fix the filament in this position. After closing the neck incision and topical application of bupivacaine (1%) and a subcutaneous 0.9% NaCl injection, the mouse was allowed to recover in a tempered (30 °C) cage, and ischemia was confirmed by checking for neurological deficits like circling behavior or tipping over onto one side. Only animals showing neuronal deficits during tMCAO were included in the study (BL6 vs. UCP2^−/−^).

MCA occlusion was kept for 60 min. Afterwards, the mouse was re-anesthetized and the filament withdrawn. The skin was closed with a surgical suture, again treated with bupivacaine, and 0.9% NaCl was injected subcutaneously for rehydration. For additional pain relief, the drinking water was supplemented with metamizole (200 mg/kg p.o.). After a recovery time in the tempered cage, the mouse was assessed by a neurological score (for more details, see Section 2.4).

In our hands, mortality rates only differed between young and aged animals (young: 4.5%, aged: 21%, *p* = 0.008, Pearson’s chi-squared test) but not between strains (*p* = 0.230) or within age groups (6 months: *p* = 0.091, 18 months: *p* = 0.737, Supplementary Table S2).

### 2.4. Neurological Scoring

Neurological assessments were performed in a blinded manner one to two hours after reperfusion and repeated 24 h later, utilizing the Longa scoring system [37]. This five-point scale quantifies the animal’s motor and reflex functions as follows: 0 = no deficit; 1 = incomplete extension of the left forepaw, indicating a mild focal deficit; 2 = leftward circling, representing a moderate focal deficit; and 3 = leftward falling, denoting a severe focal deficit. Mice scoring 4 displayed no spontaneous locomotion and exhibited reduced consciousness.

### 2.5. Gene Expression Analysis

Twenty-four hours after transient occlusion, brain slices of the ipsilateral (within the area of infarction) and contralateral areas were prepared, snap-frozen, and stored at −80 °C for further analysis of gene expression. Tissue samples were subjected to TRIzol Reagent (Invitrogen, Carlsbad, CA, USA), and the RNA isolation procedure was performed according to the manufacturer’s instructions. In the next step, any traces of genomic DNA were removed using the DNA-free™ DNA Removal Kit (Invitrogen).

For cDNA synthesis, all reagents used were from Promega Corporation (Madison, WI, USA). For a total volume of 25 µL, 1 µg RNA was reverse-transcribed into cDNA by means of Moloney Murine Leukemia Virus Reverse Transcriptase, RNase H Minus, Point Mutant (200 U) and RNasin Plus RNase inhibitor (25 U) in the presence of random hexamers (0.25 μg) and dNTP Mix (0.4 mM each). Initially, the random hexamers and the RNA were incubated for 5 min at 70 °C. The following sequence was 10 min at 20 °C and 50 min at 40 °C, followed by 15 min at 70 °C. All synthesized cDNAs were quantified and stored at −80 °C until further usage.

Target cDNA levels were analyzed by quantitative real-time PCR using AceQ qPCR SYBR^®^ Green Master Mix (Absource Diagnostics, Munich, Germany) and the following QuantiTect^®^ Primer Assays (QIAGEN, Hilden, Germany) in a qTOWER3 detection system (Analytik Jena AG, Jena, Germany): Mm_Cat_1_SG (catalase), Mm_Sod1_1_SG (Superoxide Dismutase 1), Mm_Sod2_1_SG (Superoxide Dismutase 2), Mm_Gpx1_2_SG (Glutathione peroxidase 1, Mm_Ucp2_1_SG (Uncoupling protein 2), Mm_Gapdh_3_SG (Glyceraldehyde-3-Phosphate Dehydrogenase (GAPDH), and Mm_Rn18s_3_SG (RNA18S5 (Rn18s)).

GAPDH and Rn18s served as housekeeping gene controls. All data were analyzed for both housekeeping genes. In the current manuscript, the data based on *Gapdh* are presented. PCR was started with an initial denaturation at 95 °C for 5 min, followed by 40 cycles of 10 s at 95 °C and 30 s at 60 °C. Here, the data are presented based on *Gapdh* expression. The relative expression of each mRNA compared with *Gapdh* was calculated according to the equation ΔCt = Ct_target_ − Ct_Gapdh_. After averaging at least two technical replicates, the amount of target mRNA was expressed as 2^−(ΔCt)^. The data are presented as percentage of contralateral (non-stroke-hemisphere) gene expression of BL6 mice.

### 2.6. Infarct Volume Determination

We used TTC (2,3,5-triphenyltetrazolium chloride) staining to determine infarct volume. For this, after deep anesthesia with isoflurane, mice were decapitated and the brain was rapidly removed and submerged into oxygenated ice-cold dissection solution (87 mM NaCl, 25 mM NaHCO_3_, 2.5 mM KCl, 1.25 mM NaH_2_PO_4_, 0.5 mM CaCl_2_, 7 mM MgCl_2_, 10 mM D-glucose and 75 mM sucrose; gassed with 95% O_2_, 5% CO_2_; pH 7.4; osmolality 326–328 mosmol/kg). Subsequently, 500-µm-thick coronal slices of the whole mouse brain were prepared in this ice-cold dissection solution with a vibratome (Ci7000 smz, Campden Instruments Ltd., Loughborough, UK). For staining, slices were immersed in a 2% TTC solution (in 1X Phosphate-Buffered Saline (PBS)) for 15 min at 37 °C, followed by three washes in ice-cold PBS. Afterwards, slices were fixed in 3.7% paraformaldehyde and documented by digital photography. Samples were blinded, and quantification of the infarct size was performed by a second investigator with ImageJ (Version 1.54g) depending on staining color intensity. Biological and procedural variance as assessed by infarct size variance did not differ between BL6 and UCP2^−/−^ cohorts at the age of 6 months (*p* = 0.356) and 18 months (*p* = 0.172, F-test for both analyses).

### 2.7. Slice Preparation for Electrophysiological Analysis

Mice were deeply anesthetized using isoflurane and subsequently euthanized by decapitation. The brain was quickly extracted and transferred into ice-cold, oxygenated dissection solution prepared as described above. In this solution, horizontal slices (400 µm) encompassing the hippocampus were prepared. The hippocampal formations of both hemispheres were carefully excised and then transferred into a holding chamber filled with oxygenated artificial cerebrospinal fluid (aCSF; 124 mM NaCl, 26 mM NaHCO_3_, 3 mM KCl, 1.25 mM NaH_2_PO_4_, 2.5 mM CaCl_2_, 1.3 mM MgCl_2_, 10 mM D-glucose; continuously equilibrated with carbogen (95% O_2_, 5% CO_2_); adjusted to pH 7.4; and maintained at an osmolality 304–312 mosmol/kg) at room temperature.

### 2.8. In Vitro Oxygen-Glucose Deprivation and Electrophysiological Recordings

Before starting electrophysiological experiments, hippocampal slices were allowed to recover at room temperature for at least 1 h. For electrophysiological recordings, hippocampal slices were transferred into an interface chamber maintained at 32 °C and superfused with aCSF. Field excitatory postsynaptic potentials (fEPSPs) were recorded using borosilicate glass pipettes filled with aCSF. Stimulating and recording electrodes were placed into the CA1 stratum radiatum in order to study the Schaffer collateral-CA1 synapse. The experimental design of the electrophysiological recording procedure is illustrated in Figure 3A. After habituation of the slices for at least 30 min, input/output (I/O) curves of the fEPSPs were recorded at increasing stimulation intensities (0, 20, 40, 60, 80, 100, 120, and 140 μA) with 2 frames for each stimulation strength (referred to as input/output curve 1). No significant differences in the half-maximal strength of stimulation between all four groups were found (*p* = 0.827, Kruskal-Wallis test, Supplementary Figure S1). The median stimulation strength for the following experiments was 60 µA. After that, the afferent fibers were stimulated at once per minute with a single-pulse stimulation strength adjusted to half of the maximum fEPSP amplitude.

An initial baseline recording of 10 min was completed to measure the baseline condition prior to oxygen-glucose deprivation (OGD). Next, OGD was induced via delivering aCSF without glucose and equilibrated with a 95% N_2_/5% CO_2_ gas mixture for a total of 9 min. Control experiments under standard gas mixture underwent the same protocol, but during OGD, perfusion was maintained with standard aCSF containing 10 mM glucose and a normal carbogen gas mixture. Following a 20 min recovery period, fEPSPs (at the same baseline stimulation strength) and input/output (I/O) curves were recorded again. During input/output curve 2, double-pulse stimulation (interstimulus interval of 40 ms) was used once per minute, and the paired-pulse ratio was calculated as the ratio of the 2nd pulse-evoked fEPSP to the 1st pulse-evoked fEPSP.

To investigate the effects of genipin on synaptic transmission, slices were preincubated with the UCP2 inhibitor (50 µM) or with the solvent (DMSO, final concentration 0.45% *v*/*v*) for at least 1 h in aCSF prior to administration to the interface chamber and during field potential recordings.

Only one section per mouse per condition was used for each of the four experimental groups. Thus, the statistical analysis could be based on animals afterwards.

### 2.9. Statistical Analysis

Statistical analyses were performed with IBM SPSS Statistics (Version 29.0.1.1, IBM, Ehningen, Germany). The results are presented as mean ± standard error of the mean (SEM) for the indicated number of experiments. Before statistical comparison, data were tested for normal distribution and then analyzed using tests as indicated. Mean group differences were tested for significance using the nonparametric Mann-Whitney U test. Mean differences for comparison of more than two groups were tested for significance using the nonparametric Kruskal–Wallis test before multiple comparisons subgroups were tested with post hoc Dunn’s test or with a parametric one-way analysis of variance (ANOVA, with Bonferroni-corrected *t*-test). A one-way repeated-measures ANOVA (Bonferroni *t*-test) was used to compare the animals’ performance on distance and latency in the Morris Water Maze trials. For analysis of the Morris Water Maze probe trial without the hidden platform, a two-way ANOVA (Bonferroni *t*-test) was calculated. To compare BL6 and UCP2^−/−^ mice at both age points, a two-way ANOVA (Bonferroni *t*-test) was performed. An F-test was used for variance analysis of the infarct sizes. Differences in mortality rates were assessed using Pearson’s chi-squared test. For the analysis of lifespan, a log-rank test was used. A significance level of *p* < 0.05 was considered to be statistically significant.

## 3. Results

### 3.1. Reduced Survival and Cognitive Impairment of Ucp2^−/−^ Mice

**Figure 1:**
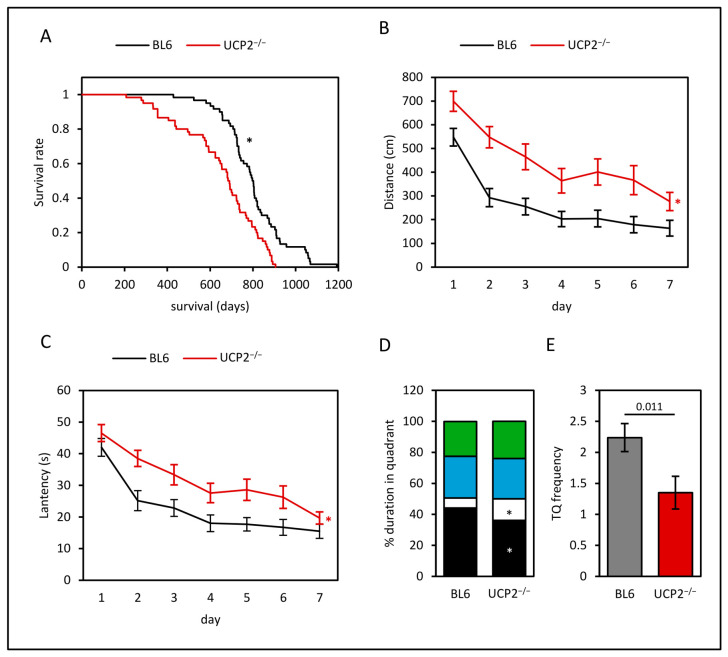
Lifespan analysis and cognitive assessment of BL6 and UCP2^−/−^ mice. (**A**) Survival, shown as Kaplan-Meier-plot, was decreased in UCP2^−/−^ mice (red, *n* = 60) compared to BL6 (black, *n* = 60), log-rank test (* *p* < 0.001). (**B**,**C**) Mice at the age of 3–6 months of the BL6 (*n* = 19) and UCP2^−/−^ (*n* = 20) strains were challenged in the Morris Water Maze for seven consecutively days. The data represent means ± SEM of (**B**) the distance and (**C**) the latency to reach the hidden platform (one-way repeated-measures ANOVA (Bonferroni *t*-test), distance: *p* < 0.001, latency: *p* = 0.002). (**D**) In the probe trial, UCP2^−/−^ mice spent less time in the target quadrant (*p* = 0.022, black-filled portion) than BL6 mice. In contrast, the duration of UCP2^−/−^ mice in the opposite quadrant (*p* = 0.033, marked in white) was found to be longer than in BL6 animals (two-way ANOVA with Bonferroni *t*-test). No significant differences for the right (green-filled portion) and left quadrant (blue-filled portion) were determined (**E**) Target quadrant (TQ) crossing in the 30-s probe trials (Mann-Whitney U test).

In initial experiments, UCP2^−/−^ mice were characterized for the phenotype focusing on lifespan and cognitive function. Therefore, a total of 60 mice (19 females and 41 males) of the control strain BL6 and 60 UCP2^−/−^ mice (34 females and 26 males) were included in a lifespan analysis (Table 1). In our hands, UCP2^−/−^ mice exhibited a reduced lifespan compared with the control cohort (Figure 1A, *p* < 0.001, log-rank test). Additionally, an intrasex comparison revealed that the lifespan reduction affects both sexes (Table 1, females (*p* < 0.001) and males (*p* = 0.025, log-rank test).

Next, cognitive function was assessed by Morris Water Maze trials for seven consecutive days. In comparison to the control strain, animals of the UCP2^−/−^ strain took a longer swimming distance in the maze to reach the hidden platform (Figure 1B, *p* < 0.001, one-way repeated-measures ANOVA with Bonferroni *t*-test). This longer distance also resulted in a prolonged latency of UCP2^−/−^ mice to finish the trial (Figure 1C, *p* = 0.002, one-way repeated-measures ANOVA with Bonferroni *t*-test). After completing the trials, the next day, the animals were challenged in two 30-s probe trials without a hidden platform in the basin. Here, BL6 mice spent more time in the target quadrant (*p* = 0.022) and less time in the opposite quadrant (*p* = 0.033, Figure 1D) than the knockout animals (two-way ANOVA). In line with this observation, the BL6 mice more often crossed the target quadrant (Figure 1E, *p* = 0.011, U test).

### 3.2. Effects of UCP2-Knockout on Ischemic Infarct Volume and Response to Oxidative Stress

**Figure 2:**
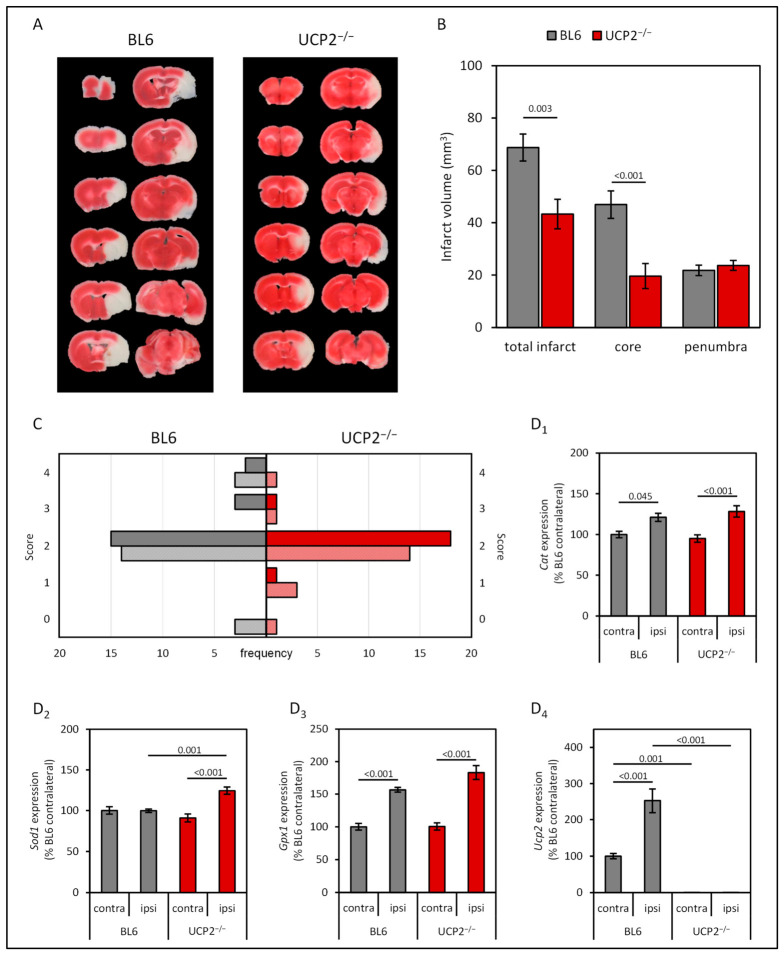
Infarct volumes after tMCAO of 6-month-old mice and expression of key players in the cellular antioxidative defense system. (**A**) Areas of the total infarct, core, and penumbra were measured 24 h after 60 min of transient MCAO by TTC staining. (**B**) The volumes are given as mean ± SEM based on *n* = 20 mice per strain (Mann-Whitney U test). (**C**) Neurological assessment based on the Longa score. The scores do not differ significantly between BL6 and UCP2^−/−^ mice after 1 h (filled bars) and 24 h (striped bars, *n* = 20 per strain, Mann-Whitney U test for comparison of the strains at both time points). (**D_1_**–**D_4_**) Expression of genes associated with oxidative stress 24 h after infarction (one-way analysis of variance (with Bonferroni-corrected *t*-test). For each group, *n* = 10 animals were analyzed. The data are illustrated as mean ± SEM of the contralateral (contra) and ipsilateral (ipsi) infarct region.

Since we found a clearly impaired phenotype of UCP2^−/−^ mice with reduced lifespan and a decline in cognitive function, we used this strain as a preclinical model for premature aging to investigate cerebral ischemic stroke. For this purpose, we challenged the animals in a reperfusion model of ischemic stroke by transient occlusion of the middle cerebral artery (tMCAO). In our model, perfusion was restricted for 60 min. Determining the infarct size after 24 h by TTC staining, UCP2^−/−^ mice exhibited a smaller total infarct volume in comparison to animals of the BL6 strain (Figure 2A,B, *p* = 0.003, U test). Since no difference in the volume of the penumbra was found (*p* = 0.525), the diminishing effect was based primarily on a reduced core volume (Figure 2B, *p* < 0.001, U test). Sex had no effect on the volume of infarction (Supplementary Figure S2).

Additionally, the mice were neurologically assessed one hour and 24 h after tMCAO using the Longa scoring system to assess locomotor impairments. It was hypothesized that UCP2^−/−^ mice would be less severely affected than control mice with a larger infarct. However, no significant differences were found between the two strains after one hour or 24 h (*p* = 0.192, respectively, *p* = 0.718, U-test, Figure 2C).

Oxidative stress is an important factor in reperfusion injury [6]. In a previous study, we have shown that UCP2^−/−^ at the age of three months exhibited higher levels of mitochondrial superoxide as a surrogate for oxidative stress [33]. In the current study, genes whose protein products are highly involved in the defense against reactive oxygen species (ROS) were analyzed in the ipsilateral and contralateral areas of the infarct volumes of BL6 and UCP2^−/−^ mice. In both strains, *Cat* (encoding for catalase) was found to be upregulated in the ipsilateral hemisphere after infarction (Figure 2(D_1_), *p* = 0.045 (BL6), respectively *p* ≤ 0.001 (UCP2^−/−^), one-way analysis of variance with Bonferroni-corrected *t*-test). Furthermore, superoxide dismutase 1 and 2 expressions were analyzed. In UCP2^−/−^, *Sod1* was upregulated in the ipsilateral hemisphere in comparison to the ipsilateral hemisphere of BL6 (*p* = 0.001) and also in comparison to the contralateral hemisphere of UCP2^−/−^ (Figure 2(D_2_), *p* < 0.001). Remarkably, no significant difference in the expression of *Sod2* was found (Supplementary Figure S3). Another important antioxidant enzyme is the glutathione peroxidase 1 (encoded by *Gpx1*). Like the catalase, transcription of *Gpx1* was found to be elevated after infarction in both strains in the ipsilateral hemisphere (Figure 2(D_3_), *p* < 0.001 for both strains). Interestingly, *Ucp2* expression was also highly induced after reperfusion to encounter oxidative stress in BL6 mice (Figure 2(D_4_), *p* < 0.001).

**Figure 3:**
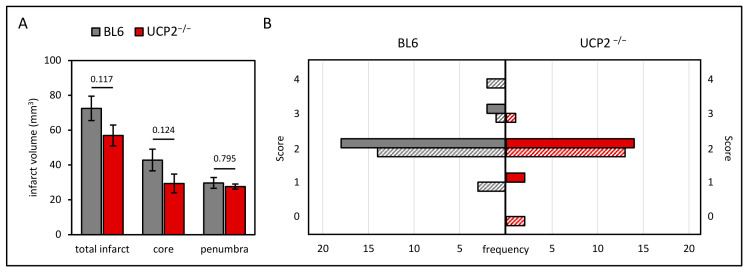
Infarct volumes and neurological scoring after tMCAO of 18-month-old mice. (**A**) Areas of the total infarct, core, and penumbra were quantified 24 h after 60 min of tMCAO. The volumes are given as mean ± SEM based on *n* = 20 (BL6) and *n* = 17 (UCP2^−/−^) mice. No significant differences were detected (Mann-Whitney U test). (**B**) Neurological assessment based on the Longa score 1 h (filled bars) and 24 h (striped bars) after infarction (BL6: *n* = 20, UCP2^−/−^: *n* = 17, no significant differences were determined, Mann-Whitney U test for comparison of the strains at both time points).

Given that our findings in six-month-old cohorts showed an infarct reduction in UCP2^−/−^ mice, we extended our analysis to a clinically more relevant age group. To this end, a small cohort of each strain was selected for tMCAO at the age of 18 months. Although the infarct size appears to be smaller in aged UCP2^−/−^ mice as well (total infarct volume: BL6 72.5 ± 6.9 mm^3^, UCP2^−/−^ 56.9 ± 6.0 mm^3^, *p* = 0.117, core: BL6 42.8 ± 6.2 mm^3^, UCP2^−/−^ 29.4 ± 5.4 mm^3^, *p* = 0.124), no significant differences in infarct volumes were observed between the two strains (Figure 3A). Moreover, no differences between sexes were found (Supplementary Figure S4, Mann-Whitney U test). As observed in the 6-month cohort, no significant differences between the two strains (1 h: *p* = 0.051, 24 h: *p* = 0.615) in the Longa score were found (Figure 3B).

Next, we tested whether age was associated with total infarct size. A two-way ANOVA with strain (BL6 vs. UCP2^−/−^) and age (6 months vs. 18 months) followed by Bonferroni post hoc testing showed that UCP2^−/−^ mice had a significantly smaller total infarct size (*p* = 0.001), whereas no significant difference was observed between the two age groups (*p* = 0.152).

### 3.3. Preserved Synaptic Transmission by the UCP2 Inhibitor Genipin in an Ex Vivo Model of Ischemic Reperfusion

**Figure 4:**
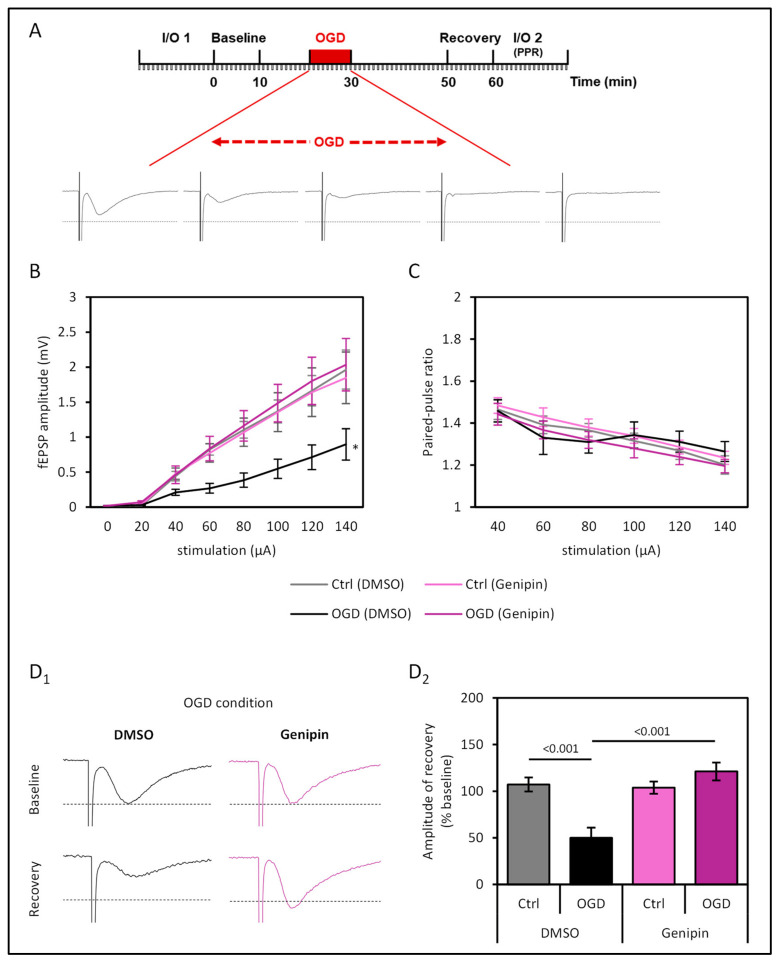
Electrophysiological characterization of genipin effects on synaptic transmission. (**A**) Experimental design of the oxygen-glucose deprivation (OGD) procedure. In the experiments, UCP2 was inhibited by adding genipin to the aCSF solution to a final concentration of 50 µmol/L. OGD conditions were limited to 9 min. For each of the four experimental groups, *n* = 10 separate experiments were performed. The data are presented as mean ± SEM. The dotted line indicates the baseline amplitude. (**B**) Input/output relations of standard gas mixture (Ctrl, 95% O_2_/5% CO_2_) and OGD-exposed cohorts at the end of the experiment. In each group, the slices were exposed to DMSO or genipin (* *p* ≤ 0.001 of DMSO-treated and OGD-stimulated vs. all other groups, Kruskal-Wallis test with post hoc Dunn’s test). (**C**) Paired-pulse ratio after OGD or standard gas mixture (95% O_2_/5% CO_2_) experiments. No significant differences were detected (Kruskal-Wallis test with post hoc Dunn’s test). (**D_1_**) Sample traces of baseline and recovery fEPSPs of OGD-exposed slices. The dotted lines indicate the baseline amplitude. (**D_2_**) Ratios of fEPSP amplitudes of recovery to baseline section of all four experimental groups were calculated (*p*-values of significant differences are given in the diagram, Kruskal-Wallis test with post hoc Dunn’s test).

Since we found an infarct-reducing effect in vivo by a knockout of *Ucp2*, we investigated the mechanism of UCP2-dependent protection in more detail. Blood flow analyses were not performed during the transient MCAO, so it was important to rule out that the observed effect in the in vivo model was not due to varying success rates in blood flow reduction. Therefore, we utilized an OGD model based on brain slices. This enabled us to determine, independent of blood flow rates, whether the inhibition of UCP2 has a beneficial effect on maintaining synaptic transmission following OGD. BL6 brain slices were challenged with genipin, a well-established UCP2 inhibitor, and postsynaptic fEPSPs were recorded in the hippocampal formation (Figure 4A).

After exposure of the brain slices to OGD conditions, input-output relations were determined. Here, a decline in the input-output relation of OGD-exposed slices compared to slices under standard gas mixture was detected (Figure 4B). Under standard gas mixture, a treatment of the slices with genipin had no effect on the input-output curves compared to slices exposed to the solvent DMSO. Remarkably, supplementing genipin to slices that were challenged to OGD prevented the decline of the input-output characteristics (*p* ≤ 0.001, Kruskal-Wallis test with post hoc Dunn’s test). Additionally, the paired-pulse ratio was calculated. No significant differences between the four experimental groups were found (Figure 4C, *p* = 0.337, Kruskal-Wallis test). Furthermore, we analyzed the fEPSP characteristics 20 min after OGD (Figure 4(D_1_), Supplementary Figure S5), and compared those data to baseline recordings at the beginning of the experimental procedure. As illustrated in Figure 4(D_2_), in OGD-challenged slices, the amplitude ratios of fEPSPs were reduced (*p* < 0.001). However, the inhibition of UCP2 by genipin prevented the decline of the amplitude (*p* < 0.001), and levels of controls with standard carbogen were reached (*p* = 1.0, Kruskal-Wallis test with post hoc Dunn’s test).

## 4. Discussion

The aim of this study was to investigate the impact of UCP2 on ischemic stroke in both ex vivo and in vivo settings. Therefore, in vivo, transient MCA occlusion and, ex vivo, oxygen-glucose deprivation served as preclinical stroke models. We further hypothesized that inhibition of UCP2 would preserve synaptic function under ischemia-like conditions.

To establish the UCP2^−/−^ model in our lab, a baseline characterization was performed with respect to lifespan and cognitive function, which served as a surrogate for a neuropathological phenotype. The missing proton leak in UCP2^−/−^ mice via a functional anion carrier may increase oxidative stress, which may eventually contribute to variation in murine lifespan. We confirmed the observation of a reduced lifespan in UCP2^−/−^ mice, affecting both sexes [33,38]. Since UCP2^−/−^ mice lack a major regulator of ROS homeostasis, the reduced lifespan was associated with increased oxidative stress in organs, including the brain. Moreover, in transgenic models of UCP2 overexpression, extended lifespans in mice and flies were observed [39,40]. These findings are in line with observations that conplastic mouse strains, harboring genetic alterations in OXPHOS genes that confer oxidative stress, have a reduced lifespan [41]. However, diametral effects with a prolonged lifespan were found [34]. For the first time, we have shown that UCP2^−/−^ mice exhibit impaired cognition based on spatial learning performance in the Morris Water Maze, complementing findings of open-field and Y-maze studies [19,42].

One major finding of our study was that a UCP2 knockout at the age of 6 months resulted in a reduced infarct volume after transient occlusion of the middle cerebral artery, mainly restricted to the core of the lesion, whereas at 18 months, no significant differences between the two groups were found. However, based on analysis employing a two-way ANOVA, no significant difference in the factor age was calculated. UCP2^−/−^ exhibits an accelerated aging process [33], and a reduced lifespan. Therefore, we expect a much higher biological variance in the aged group of UCP2^−/−^. As a consequence, a much higher number of animals would be needed to reveal a significant difference in the size of infarction. This does not exclude beneficial effects of the knockout regarding the severity of infarction.

So far, the impact of a UCP2 knockout on ischemic stroke is inconsistent, depending on the model and experimental conditions. Consistent with our findings, in a model of permanent occlusion, a UCP2 knockout contributed to a decreased size of infarction [23]. In contrast, the majority of the studies reported increased infarct volumes after transient MCAO in UCP2^−/−^ mice compared to control strains [20,21,22]. Additionally, overexpression of human UCP2 in mice reduced infarct size [43,44], whereas downregulation of UCP2 in the SHRSP rat strain increased susceptibility to stroke [45]. What could contribute to the different observations? There are two principal differences between our investigation and the previous studies.

One factor could be the age of the mice at the time point of infarction. Since Mattiasson et al. 2003 and Haines et al. 2010 did not provide information on age, those data could not be included in the discussion [20,44]. However, in all other studies, the age of mice was 2–3 months [21,22,23], at least 3 to 4 months younger than the animals used in our study. Since one major factor of ischemia-reperfusion injury is ROS, it is of particular importance that at the age of 3 months, UCP2^−/−^ mice exhibit higher ROS levels in the hippocampus than wild-type animals, but those differences were not detectable in mice at the age of 6 months or older [33]. In line with the concept of mitohormesis [28,46], mild oxidative stress prior to ischemia-reperfusion injury may help to bear the burden and thereby reduce infarct volume. Interestingly, we did not find a differential expression of antioxidant players in the contralateral hemisphere between the two cohorts. Instead, in both strains, the response to the occlusion resulted in an upregulation of those enzymes. Hence, we could not exclude differential enzyme activity as an early response, accompanying the ischemia-induced upregulation of antioxidants that was determined after 24 h [47].

With respect to UCP2, in contrast to our findings, a decreased expression of the anion carrier was reported after hypoxia based on MCAO or OGD conditions [22]. Furthermore, UCP2 deficiency was linked to enlarged infarct volumes and aggravated neurological deficit scores in mice. However, in rats expressing human UCP2, ischemia led to an increased expression of the anion carrier [44]. The level of UCP2 expression seems to be dependent on hypoxic conditions and tissue. After ischemia and reperfusion, in the kidney, UCP2 was upregulated [48], whereas in cardiomyocytes, expression varies with the severity of hypoxia [49]. In an ex vivo OGD model, UCP2 protein expression was increased [26].

In contrast to the mitohormetic hypothesis, the infarct-reducing effect of the absence of functional Ucp2 carriers was not significant in the cohorts of 18-month-old animals.

One might speculate here that this lost effect is due to greater variability in aged animals, and therefore, a larger number of mice would simply have been required. Or whether during the lifetime, detrimental effects of the knockout on physiological functions prevail and, instead, the animals suffer from an accelerated aging process [33]. In a model of acute pancreatitis, UCP2^−/−^ mice at the age of 3 months were indistinguishable from age-matched controls, whereas at the age of 12 months, wild-type mice were less prone to pancreatic damage than animals harboring the knockout [50].

Another factor that may contribute to the differential findings between the hitherto existing studies could be the sex of the animals. In our experiments, we used both female and male mice, unlike previous studies, in which only male mice were used. However, no significant differences between female and male mice with respect to infarct sizes were determined in our study. Altogether, the data indicated that age and preconditioning of the hypoperfused tissue critically influence the outcome of UCP2 modulation in ischemic injury.

The reduction in infarct size did not result in improved motor and behavioral performance, as assessed by the well-established Longa scoring system. Thus, the Longa score may have been a too-insensitive measure to detect subtle improvements in mice. While the Longa score is mainly based on spontaneous observations, more complex tests challenging the animals with respect to motor functions or reflexes, such as the modified neurological severity score (mNSS) [51], might have helped to elucidate potential benefits of the UCP2 knockout after 60 min of tMCAO [52].

The knockout of UCP2 is a feasible preclinical model of oxidative stress [11], but it is limited in its translation to the clinical situation, since UCP2 alterations are scarce. So far, a number of polymorphisms were identified that affect obesity and diabetes [53]. Therefore, the concept of a UCP2-dependent perturbation was extended to experiments based on the proton carrier inhibition by genipin, a herbal non-glycoside iridoid [54].

One key finding of our study was that the neuroprotective effect of genipin was mediated by the preservation of synaptic integrity in the hippocampal formation. While oral administration of genipin to C57BL/6 mice reduced infarction volume [26], the molecular mechanisms of neuroprotection remain incompletely understood [55].

The neuroprotective effect of genipin may not be limited to UCP2 inhibition alone [54]. In oncological in vitro studies, genipin has also been reported to attenuate signaling pathways such as sonic hedgehog and STAT3 [56,57,58]. Notably, STAT3 activation has been linked to UCP2 expression [59], and vice versa, UCP2 expression under oxidative stress has been shown to depend on functional STAT3 [60]. In addition, several studies reported genipin as a mediator of antioxidant activity, which is consistent with its role as a suppressor of oxidative stress (summarized in Ref. [54]). Based on the current knowledge, we cannot exclude that mechanisms other than UCP2 inhibition contribute to the preservation of synaptic transmission in the hippocampal network; however, UCP2 blockade appears to be the principal mechanism of action [24].

Under oxidative stress, genipin antagonizes mechanisms that contribute to cell death [26,61]. Quite the opposite was found in cancer models, in which genipin induces apoptosis of glioblastoma [62], neuroblastoma cells [63], and colorectal cancer [57]. Furthermore, it was also conceivable that genipin had an influence on the tMCAO model itself. For instance, loss of functional Ucp2 may perturb the calcium homeostasis of cells [64,65] that, in the end, may affect the vascular tone and thereby perfusion of the tissue. Since the hippocampal brain slices were supplied by diffusion ex vivo, our data indicate a more direct effect on the synaptic transmission.

One limitation of our study is the lack of direct ROS measurements after ischemic stroke in the brain slices. Since ROS levels were enhanced in native UCP2^−/−^ mice at the age of 3 months, but not thereafter [33], we speculated that such a ROS analysis would require large numbers of animals to detect significant strain differences after stroke. Rather, we decided to focus on determining infarct size and to store tissue for subsequent expression analysis. Here, our data demonstrate that antioxidant enzymes were upregulated in both mouse strains after infarction, indicating oxidative stress in the process of ischemia and reperfusion [6]. Only *Sod1* expression was higher in UCP2^−/−^ mice than in the control group after infarction. As we did not perform direct enzymatic activity level measurements, this only implies that superoxide levels may differ across the examined strains under ischemia reperfusion conditions.

An aspect limiting the translational impact of our results is the circumstance that electrophysiological assessment was performed only on slices exposed to genipin ex vivo. While our data demonstrate a neuroprotective effect of genipin on synaptic transmission in an acute model of hypoxia in young-adult animals, no data on a long-term in vivo treatment of genipin with a subsequent analysis of the neuronal network exist to date. Therefore, further long-term in vivo studies employing genipin or its derivatives [66] should include older animals that reflect the growing elderly population and better represent the demographic group most affected by stroke.

## 5. Conclusions

In the present study, UCP2^−/−^ mice at the age of 6 months were less prone to ischemia than the control strain, resulting in a smaller core area after tMCAO. However, this beneficial effect of the UCP2 knockout could not be consistently generalized to the cohort of old age. Pharmacological inhibition of UCP2 with genipin restored synaptic function after oxygen-glucose deprivation ex vivo, suggesting a potentially neuroprotective mechanism at the level of neuronal network integrity. Moreover, the translational relevance of these findings, particularly in vivo and in aged populations, remains to be established.

## Figures and Tables

**Table 1:** Lifespan analysis of BL6 and UCP2^−/−^ mice.

Strain	Sex	*n*	Median Survival (Days)	95% CI (Days)	Mean Survival ± SEM (Days)
BL6	female	19	820	772–868	800 ± 30
	male	41	772	709–835	805 ± 23
	total	60	797	775–819	804 ± 18
UCP2^−/−^	female	34	655	594–716	632 ± 31
	male	26	704	645–763	677 ± 37
	total	60	684	663–706	652 ± 24

## Data Availability

The original contributions presented in this study are included in the article/Supplementary Material. Further inquiries can be directed to the corresponding author.

## Supplementary Materials

The following supporting information can be downloaded at: https://www.mdpi.com/article/10.3390/cells15141299/s1, Figure S1: Input/output relations of all four groups prior to the change of the perfusion conditions to OGD; Figure S2: Infarct volumes were histologically assessed 24 h after 60 min of tMCAO in mice at the age of 6 months; Figure S3: Gene expression of Sod2 24 h after 1 h tMCAO; Figure S4: Infarct volumes were histologically assessed 24 h after 60 min of tMCAO in mice at the age of 18 months; Figure S5: Sample traces of baseline and recovery fEPSP of normoxia-exposed slices derived from BL6 mice incubated with either DMSO or genipin; Table S1: Overview of animals used in the study; Table S2: Perioperative mortality and excluded animals in the tMCAO model.

## Abbreviations

The following abbreviations are used in this manuscript:

aCSF	artificial cerebrospinal fluid
fEPSP	field excitatory postsynaptic potentials
MCAO	middle cerebral artery occlusion
ROS	reactive oxygen species
TG	target quadrant
TTC	2,3,5-triphenyltetrazolium chloride
UCP2	uncoupling protein 2
