# Supplementary material for: Reduced Cerebral Infarct Volume in Young UCP2−/− Mice and Preserved Synaptic Transmission by Genipin
Source: Cells. 2026 Jul 21;15(14):1299. doi: 10.3390/cells15141299 (PMC13406320; doi:10.3390/cells15141299)
Supplement: Supplementary file 1 [file cells-15-01299-s001.zip › cells-4402662-supplementary.pdf]

Supplement

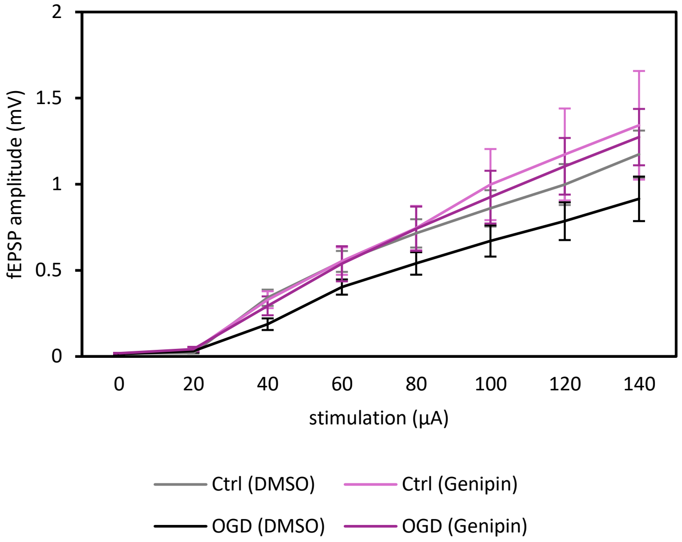

Supplementary Figure S1: Input/output relations of all four groups prior to the change of the perfusion conditions to OGD. In each group, the slices were exposed to DMSO or genipin ($p = 0.827$; Kruskal-Wallis test with post hoc Dunn's test). For each group, $n = 10$ animals were analyzed. The data are illustrated as mean ± SEM. Ctrl: Control conditions with standard carbogen

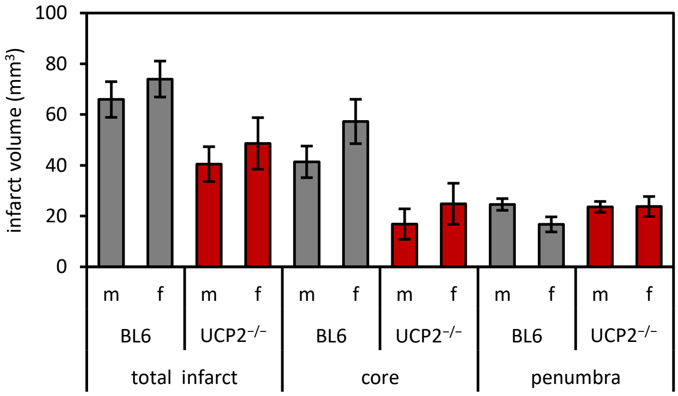

Supplementary Figure S2: Infarct volumes were histologically assessed 24 hours after a tMCAO for 1 hour in mice at the age of 6 months. The volumes are given as mean ± SEM based on $n = 7$ female (f) and $n = 13$ male (m) mice per strain. No significant differences were detected between the sexes (Mann-Whitney U test).

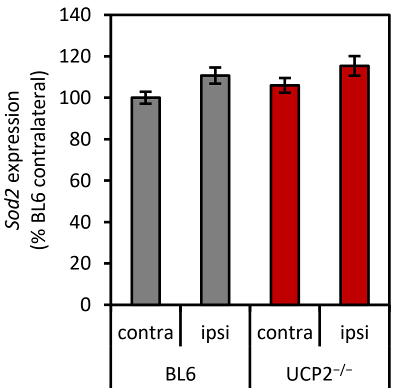

Supplementary Figure S3: Gene expression of *Sod2* 24 hours after 1 hour tMCAO. No significant differences were detected (One-Way Analysis of Variance (with Bonferroni-corrected t-test). For each group, $n = 10$ animals were analyzed. The data are illustrated as mean ± SEM of contralateral (contra) and ipsilateral (ipsi) hemispheres.

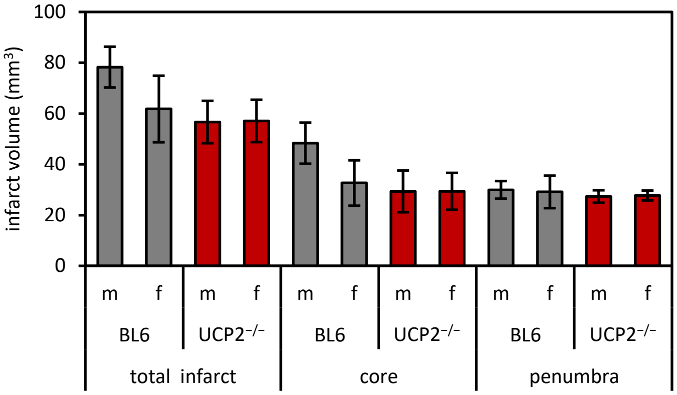

Supplementary Figure S4: Infarct volumes were histologically assessed 24 hours after a tMCAO for 1 hour in mice at the age of 18 months. The volumes are given as mean ± SEM based on the following number of animals: BL6 (n = 7 female (f), n = 13 male (m) UCP2$^{-/-}$ (n = 6 female, n = 11 male). No significant differences were detected between the sexes (Mann-Whitney U test).

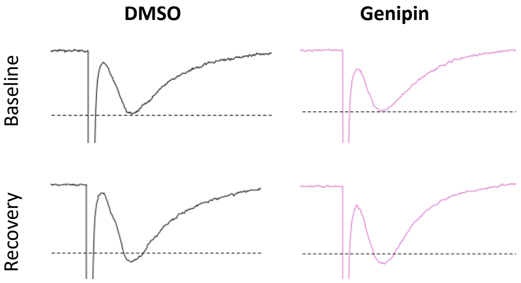

Supplementary Figure S5: Sample traces of baseline and recovery fEPSP of normoxia-exposed slices derived from BL6 mice.

Supplementary Table S1: Overview of animals used in the study.

Method	BL6	UCP2$^{-/-}$
Lifespan analysis (including 20 from Morris Water Maze analysis)	60	60
Morris Water Maze	20	20
	**tMCAO (6 months)**	
Infarct volume and neuro score	24 (20 analyzed)	21 (20 analyzed)
Gene expression	10	12 (10 analyzed)
	**tMCAO (18 months)**	
Infarct volume and neuro score	26 (20 analyzed)	21 (17 analyzed)
Electrophysiological recordings	18	
**Total number of animals:**	138	114

Supplementary Table S2 : Perioperative mortality and excluded animals in the tMCAO model.

Experimental group	Number of animals
BL6<br>(6-month-old)	30 mice analyzed (infarct volume and neuro score, gene expression)<br>3 animals died during the tMCAO procedure (= 9% mortality)<br>1 mouse was excluded due to vascular malformation<br>= 88% could be used for data acquisition
UCP2$^{-/-}$<br>(6-month-old)	30 mice analyzed (infarct volume and neuro score, gene expression)<br>no animal died during the tMCAO procedure (= 0% mortality)<br>1 mouse was excluded due to problems during anesthetization<br>2 mice were excluded due to heavy bleeding<br>= 91% could be used for data acquisition
BL6<br>(18-month-old)	20 mice analyzed (infarct volume and neuro score)<br>6 animals died during the tMCAO procedure (= 23% mortality)<br>= 77% could be used for data acquisition
UCP2$^{-/-}$<br>(18-month-old)	17 mice analyzed (infarct volume and neuro score)<br>4 animals died during the tMCAO procedure (= 19% mortality)<br>= 81% could be used for data acquisition
